# Inflammatory markers and physical frailty: towards clinical application

**DOI:** 10.1186/s12979-023-00410-3

**Published:** 2024-01-06

**Authors:** Yiming Pan, Lina Ma

**Affiliations:** Department of Geriatrics, Xuanwu Hospital Capital Medical University, National Research Center for Geriatric Medicine, Beijing, 100053 China

**Keywords:** Physical frailty, Inflammation, Marker, Biological, Aging

## Abstract

Global population aging poses a tremendous burden on the health care system worldwide. Frailty is associated with decreased physical reserve and is considered an important indicator of adverse events in the older population. Therefore, there is growing interest in the early diagnosis and intervention of frailty, but the cellular mechanisms responsible for frailty are still not completely understood. Chronic inflammation is related to decreased physical function and increased disease risk. Additionally, multiple human and animal studies suggest that inflammation probably plays the largest role in contributing to frailty. Some inflammatory markers have been proposed to predict physical frailty. However, there are still large gaps in knowledge related to the clinical application of these markers in frail patients. Therefore, understanding the biological processes and identifying recognized and reliable markers are urgent and pivotal tasks for geriatricians. In the present review, we broadly summarize the inflammatory markers that may have potential diagnostic and therapeutic use, thereby translating them into health care for older people with frailty in the near future.

## Introduction

Global populations are aging rapidly, which has a major impact on the health care system. Chronic diseases lead to increased vulnerability and resistance, which are the core cause of frailty. Physical frailty is characterized by decreased functional reserve and increased vulnerability to adverse health outcomes [1, 2]. Because frailty is increasing relevant to identify patients at higher risk in clinical practice, there is a growing interest in the early diagnosis and prevention of frailty [3]. However, to date, of all potential biological etiologies, there is still a lack of recognized, accurate and reliable biological markers for frailty. High levels of inflammatory markers are common in older adults, and can predict the risk for frailty, decreased physical function, and comorbidities [4]. Low-grade chronic inflammation (CI) stands out in that serum inflammatory markers change early at the stage between cellular abnormalities and systems dysfunction [5], furthermore, there is evidence that midlife systematic CI was independently associated with increased frailty risk in later life [6]. Thus, identification of highly sensitive inflammatory markers for the early diagnosis and intervention of frailty may help identify important diagnostic clues.

## Search strategy

Studies research from inception until August 2023 was conducted using the PubMed database and Web of Science with the following search terms: inflammation, chronic inflammation, inflammaging, inflammatory, inflammatory index, markers, biomarkers, frailty, frail, aging, aged, and older adults. Studies written in English were included. Studies employing a non-validated frailty assessment tool, or conference abstracts were excluded.

## Association between physical frailty and chronic inflammation

Low-grade CI is a hallmark of the aging process [7]. Particularly, low-grade, chronic, systemic inflammation is 2- to 3-fold elevated in elderly individuals [8], which indicates inflammation may be the molecular mechanism underlying physical frailty and inflammaging.

### Etiology of chronic inflammation

CI is associated with low-grade, persistent physiological responses and can lead to serious clinical outcomes [9]. Multiple factors such as chronic exposure to stress, obesity, and chronic periodontitis, induce biological processes including DNA damage, impaired autophagy, and elevated oxidative stress due to mitochondrial dysfunction, further lead to metabolic dysfunction, cellular senescence, and ultimately cellular necrosis, then activate innate immunity pathway, produce senescence-associated secretory phenotypes, and contribute to the releasing of proinflammatory cytokines and chemokines into circulation [4, 10–13], finally result in age-related diseases [14]. Furthermore, the damage-associated molecular patterns (DAMPs) released from damaged cells, genomic instability, changes in the gut microbiota, NOD-, LRR- and pyrin domain-containing protein 3 (NLRP3) inflammasome activation, and chronic viral infections contribute to inflammaging [15–18].

### Aging and serum markers of inflammation

As a basic defense mechanism, inflammation has positive effects on health such as resistance to external pathogens, identification and elimination of internal abnormal cells, and tissue repair. However, during aging, a defect in inflammation resolution occurs and leads to CI [19]. Current evidences indicate that aging is characterized by an increase in pro-inflammatory cytokines and a reduction in anti-inflammatory cytokines induced by age-related immune, hormonal, and adipose changes [20–22], leading to a low-grade chronic inflammatory state, which finally motivates the occurrence of chronic inflammatory diseases and frailty [4], and accelerates aging. Furthermore, higher levels of pro-inflammatory cytokines are correlated with aging and increased risks of morbidity and mortality [23, 24].

### Physical frailty and serum markers of inflammation

**Fig. 1:**
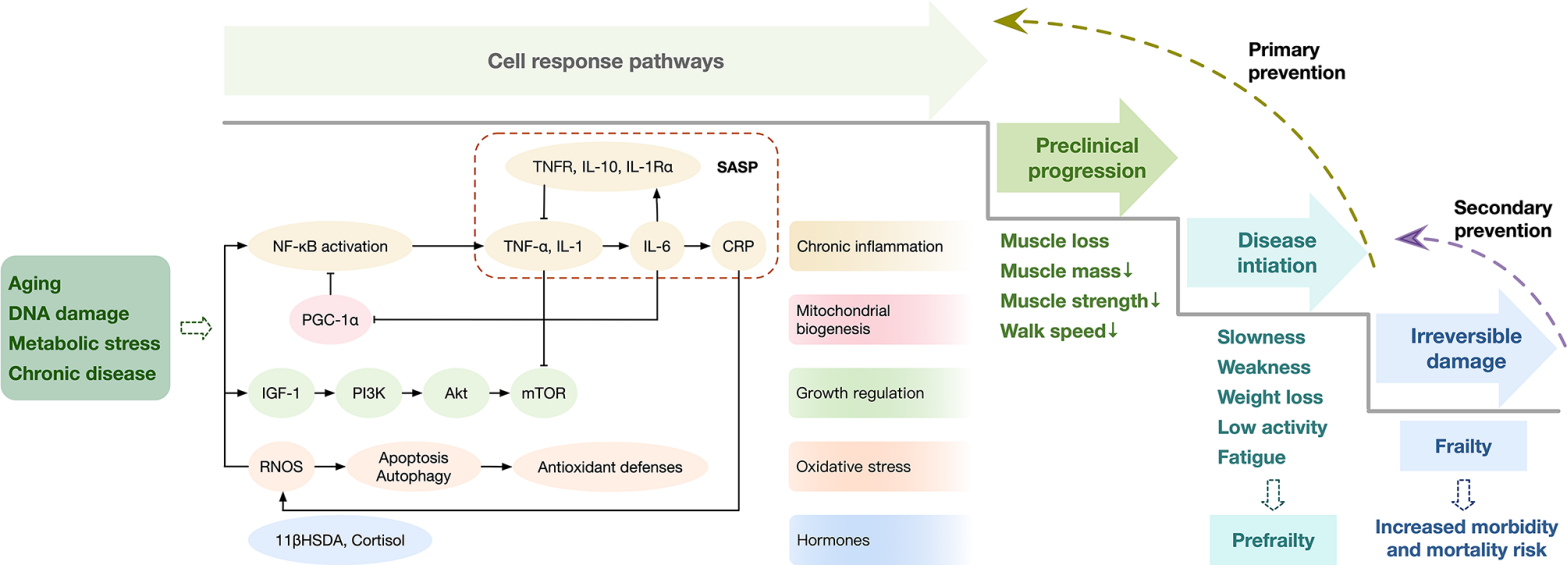
Hypothetical hierarchical model of frailty. (1) This figure shows the central role of inflammation in multiple pathways leading to frailty. Aging, DNA damage, metabolic stress and chronic diseases lead to a systematic inflammatory response in the skeletal muscle, which induces the inflammatory signaling pathway. Additionally, other important pathways, such as the PI3K/Akt/mTOR, PGC-1α and oxidative stress pathways, are involved. Hormones and mitochondria also contribute pathogenically to frailty. Inflammation probably plays the most significant role in contributing to frailty. (2) There are four stages in the frailty process: skeletal muscle physiology (cell response pathways), preclinical progression of frailty, frailty initiation and irreversible damage. In the third stage, primary prevention may reverse the process. In the fourth stage, secondary prevention may delay the progression of frailty. Biomarkers can be found in the second, third and fourth stages. The identification of earlier biomarkers will allow better prognosis through prevention and intervention in elderly patients. Abbreviations: IL, interleukin; TNF, tissue necrosis factor; CRP, c-reactive protein; SASP, senescence-associated secretory phenotype; RNOS, Reactive nitrogen oxygen species; IGF-1, Insulin-like growth factor 1; PI3K, Phosphoinositide 3-kinase; Akt, Protein kinase B; mTOR, mammalian target of rapamycin; PGC-1α, peroxisome proliferator-activated receptor-gamma coactivator − 1 α; 11βHSDα, 11β-Hydroxysteroid dehydrogenase α

Physical frailty represents a reduced ability to cope with stressors, the biological mechanisms underlying CI in frail adults have not been well understood yet. An intricate interplay between the inflammatory response, apoptosis, mitochondria, oxidative stress and autophagy might be involved in the onset of frailty [25–27]. A full understanding of these processes may lead to new therapeutic strategies for inflammatory disorders [28]. As shown in Fig. 1, many important pathways, such as the Phosphoinositide 3-kinase/Protein kinase B/mammalian target of rapamycin and peroxisome proliferator-activated receptor-γ coactivator-1α pathways, are involved. Among these, inflammation probably plays the most significant role in contributing to frailty, which may be related to the imbalance of energy catabolism and interference with homeostatic signals [4].

It is promising to selectively modulate inflammation early in health decline to slow frailty and other aging-related phenotypes that emerge with aging. Therefore, finding a biological marker with good diagnostic and prognostic capacity would be a major milestone for easily identifying pre-frail and frail status. This would allow physicians to identify frailty risks, monitor the progression and efficacy of interventions and prevent and delay the onset of frailty and occurrence of disability (Fig. 1).

## Inflammatory biomarkers for frailty

Biomarkers are an essential tool to translate scientific concepts into diagnostic and therapeutic approaches and technologies [29]. Frailty status explained a greater percentage of variability in markers of inflammation than age in older adults [30], which indicated a close association between frailty and inflammatory markers. In the past 10 years, investigation of biological markers of frailty has gained impetus, but the inflammatory markers with the most potential for clinical application have not yet been defined and need to be further investigated. Markers of inflammaging may include immune cell markers, serum cytokine markers and microRNAs [31]. Common circulating pro-inflammatory cytokines include interleukin-1 (IL-1), IL-6, IL-8, IL-13, IL-18, C-reactive protein (CRP), interferon-α (IFN-α) and IFN-β, transforming growth factor-β (TGFβ), tumor necrosis factor (TNF) and its receptors (TNFR1 and TNFR2), etc.

### Frailty definitions and measurements

Over the last decades, researchers have made great efforts in the field of frailty measurement, but until now, there still lacks a unique, standardized and universally agreed upon operational definition for physical frailty. The most frequently used criteria of frailty are based on five physical determinations described in the Cardiovascular Health Study (CHS) [32], and the prognostic capacity has been extensively validated [33]. Fried phenotype defines frailty as a syndrome with physiological etiology characterized by decreased reserve and resistance to stressors, thus can be used to elucidate mechanisms or develop targeted intervention [32]. Frailty index (FI) is the second most widely used criteria by evaluating many age-related health deficits including both psychological and social factors [34]. This cumulative deficit method was proved to be useful for prediction for mortality and other adverse health outcomes in older adults, but lack of a unifying biological theory related to aging [35]. Due to the difficulty to put into practice in busy clinical work regarding the above two measurements, many convenient alternative frailty assessment tools have emerged, such as Fatigue, Resistance, Ambulation, Illness, Loss of weight (FRAIL) scale [36], Frailty Screening Questionnaire (FSQ) [37], Edmonton Frailty Scale (EFS) [38], Groningen Frailty Indicator (GFI) [39], Tilburg Frailty Indicator (TFI) [40], and Multidimensional Prognostic Index (MPI) [41]. Of all the various measurements, frailty phenotype approach is considered to be a more useful tool towards better understanding of the biological aging and late-life vulnerability.

### Inflammatory markers of frailty according to different frailty criteria

The absence of a unified operational criteria for physical frailty and the complex underlying pathophysiology of frailty make the development of biomarkers extremely challenging. In recent years, there have been seven studies focusing on inflammatory markers of physical frailty using different criteria (Table 1). Collerton et al. found that biomarkers were generally consistent between the Fried and FI frailty models in the very old [42]. Chao et al. compared six types of questionnaire in chronic dialysis patients and found that the FRAIL scale score was significantly correlated with serum albumin levels [43]. However, the study sample size was relatively small. Lu et al. identified a number of biomarkers were associated with frailty in older adults [44]. Furthermore, they suggested that IL-6:soluble interleukin-6 receptor (sIL-6R): soluble glycoprotein 130 (sgp130) complex may be involved in the development of frailty according to both Fried and FI criteria, and sIL-2Rα was an independent risk factor for frailty based on Fried frailty phenotype [44]. The results based on factor analysis showed that the factor group consisting of IFN-γ, IL-4, IL-1β, IL-1Rα, granulocyte colony stimulating factor (G-CSF), IL-8, IL-6 and IL-7 showed correlation with FP, which was not affected by adjusting for age; but it was not related to FI [45]. Frail subjects with lung transplants tended to have higher TNF-R1 levels according to both Fried frailty phenotype and Short Physical Performance Battery (SPPB) assessment [46]. In a 12-year longitudinal study, CRP and fibrinogen were associated with longitudinal changes in FI but not with changes in Fried phenotype [47]. These results suggest that different frailty criteria have an impact on the identification of inflammatory markers, which reduces the comparability of results between different studies.

**Table 1:** Inflammatory markers of frailty according to different frailty criteria

Studies	Design	Population	Inflammation parameter of frailty using different measurements						
			FP	FI	Function	CGA	FRAIL	SF, EFS, GFI, G8, and TFI	SPPB
Hubbard 2009	Cross-sectional	> 75y, n = 110	IL-6, TNF-α, CRP, Albumin	IL-6, TNF-α, CRP, Albumin	IL-6, TNF-α, CRP, Albumin	N/S	N/S	N/S	N/S
Ronning 2010	Cross-sectional	> 70y, n = 137	IL-6, TNF-α, CRP, D-dimer	N/S	N/S	IL-6, TNF-α, CRP, D-dimer	N/S	N/S	N/S
Collerton 2012	Cross-sectional	> 85y, n = 845	IL-6, TNF-α, CRP, Albumin	IL-6, TNF-α, CRP, albumin, WBC, neutrophils	N/S	N/S	N/S	N/S	N/S
Chao 2015	Cross-sectional	~ 67.3y, n = 46	N/S	N/S	N/S	N/S	Albumin	None	N/S
Singer 2015	Nested case-control	> 18y, n = 395	IL-6, TNFR1, Leptin	N/S	N/S	N/S	N/S	N/S	TNFR1
Lu 2016	Cross-sectional	> 55y, n = 76	spg130, I-309, MCP-1, IL-6R, IL-2Ra	spg130, I-309, MCP-1, BCA-1, RANTES, Leptin	N/S	N/S	N/S	N/S	N/S
Hsu 2019	Longitudinal (3y)	> 75y, n = 901	IL-6, IL-8; Factor consisting of IFN-γ, IL-4, IL-1β, IL-1Rα, G-CSF, IL-8, IL-6 and IL-7	IL-6, IL-8	N/S	N/S	N/S	N/S	N/S
Welstead 2020	Longitudinal (12y)	~ 69.6y, n = 550	-	CRP, fibrinogen	N/S	N/S	N/S	N/S	N/S

Abbreviations: FP, frailty phenotype; IL, interleukin; TNF, tumor necrosis factor; CRP, C-reactive protein; BCA, B-cell attracting chemokines; MCP, monocyte chemoattractant protein; spg130, soluble glycoprotein 130; RANTES, regulated upon activation, normal T-cell expressed and secreted; IFN, interferon; G-CSF, granulocyte colonystimulating factor; CGA, Comprehensive Geriatric Assessment; FRAIL, Fatigue, Resistance, Ambulation, Illness, Loss of weight; SF, Strawbridge Frailty questionnaire; EFS, Edmonton Frailty Scale; GFI, Groningen Frailty Indicator; G8, G8 questionnaire; TFI, Tilburg Frailty Indicator; SPPB, Short Physical Performance Battery

### Is there a best inflammatory marker of frailty?

There are some studies focusing on the comparison between different inflammatory markers of frailty (Table 2). IL-6 was one of the first identified myokines and is related to disability and mortality [48]. As Fig. 1 shows, IL-6 has paradoxical effects with both pro-inflammatory and anti-inflammatory roles, which are most likely related to the environment and TNF-α [49]. A study using a large cohort showed that IL-6 is a reliable marker of disability [50]. Multiple cross-sectional studies have shown that elevated circulating IL-6 levels are significantly associated with frailty after adjusting for age, gender and other confounding factors in different populations [51, 52]. Gómez-Rubio et al. creatively examined the salivary IL-6 concentration in older women and analyzed its correlation with frailty, and the results were consistent with the performance of circulating IL-6 [56]. Several longitudinal studies also provide evidence for the predicted role of IL-6 in frailty, poor functional performance and mobility [53–55]. In terms of clinical application, IL-6 level can be used to distinguish different frailty phenotypes (frail, pre-frail and robust), especially suitable for frailty screening, but the exact cut-off value still needs more population-based studies [54, 56]. The mechanism of IL-6 involved in frailty may be associated with IL-6 gene variation [57] and the intercellular communication function of extracellular vesicles [58].

**Table 2:** Comparison of inflammatory markers for frailty

Studies	Design	Population		Inflammation parameter					
				TNF-α	TNFR1	TNFR2	genotype	IL-6	CRP
Schaap 2009	Longitudinal (5y)	≥ 70y, n = 2177		+	+	+	N/S	-	-
Aguirre 2014	Cross-sectional	≥ 65y, n = 107		N/S	+	N/S	N/S	-	-
Arts 2015	Cross-sectional	≥ 60y, n = 366		N/S	N/S	N/S	N/S	-	-
Liu 2016	Cross-sectional	> 60y, n = 1919		N/S	N/S	-	N/S	+	-
Mekli 2016	Cross-sectional	≥ 50y, n = 3160		N/S	N/S	N/S	+	N/S	N/S
Tay 2016	Longitudinal (1y)	≥ 65y, n = 99		+	N/S	N/S	N/S	-	N/S
Van Epps 2016	Cross-sectional	≥ 60y, n = 117		N/S	+	+	N/S	+	-
Langmann 2017	Longitudinal (2y)	≥ 65y, n = 178	Baseline	N/S	+	+	N/S	+	+
			1y Follow-up	N/S	-	-	N/S	+	+
Marcos-Pérez 2018	Cross-sectional	≥ 65y, n = 259		+	N/S	+	N/S	+	+
Yang 2018	Cross-sectional	≥ 60y, n = 435		N/S	N/S	N/S	N/S	-	-
Navarro-Martínez 2019	Cross-sectional	≥ 50y, n = 46		-	N/S	N/S	N/S	+	+
Prince 2019	Cross-sectional	≥ 30y, n = 68		N/S	N/S	N/S	+	N/S	N/S
Buigues 2020	Longitudinal (1y)	≥ 50y, n = 39	Baseline	-	N/S	N/S	N/S	+	+
			1y Follow-up	-	N/S	N/S	N/S	+	-
Furtado 2020	Cross-sectional	≥ 75y, n = 358		+	N/S	N/S	N/S	+	-
Castellana 2021	Longitudinal (6y)	≥ 65y, n = 1929		+	N/S	N/S	N/S	+	-
Castro-Herrera 2021	Cross-sectional	≥ 65y, n = 184		-	N/S	-	N/S	-	+
McKechnie 2021	Longitudinal (3y)	≥ 70y, n = 981		N/S	N/S	N/S	N/S	+	-
Teixeira-Gomes 2021	Cross-sectional	≥ 65y, n = 291		N/S	N/S	N/S	N/S	+	+
McKechnie 2022	Cross-sectional	≥ 70y, n = 1399		N/S	N/S	N/S	N/S	+	+
Pansarasa 2022	Cross-sectional	≥ 75y, n = 219		+	N/S	N/S	N/S	+	+
Samson 2022	Longitudinal (20y)	≥ 65y, n = 144	Baseline	N/S	N/S	N/S	N/S	+	+
			5y Follow-up	N/S	N/S	N/S	N/S	-	-

Abbreviations: IL, interleukin; TNF, tumor necrosis factor; CRP, C-reactive protein

As another inflammatory marker widely used in clinical practice, the association of CRP with frailty has also been extensively studied, although the results are inconsistent. Cross-sectional associations of CRP and high-sensitivity CRP (hsCRP) with frailty and pre-frailty have been demonstrated in several studies in older adults [51, 58, 59]. Furthermore, in terms of physical function, CRP and hsCRP levels were independently associated with grip strength and predictive of grip strength decline [53, 60]. A prospective study found that CI as measured by either CRP at baseline or longitudinal stable CRP was associated with higher odds of frailty 6–24 years later [6], which provides strong evidence for identifying the physiological underpinning of frailty. However, another longitudinal study with a small sample size in prostate cancer patients receiving antiandrogen therapy found that CRP did not predict frailty progression after one year [54]. There are many reasons for this discrepancy, such as subject selection, comorbidity, and frailty criteria. It should be noted that studies on the predictive effect of CRP on frailty are limited, and there is a lack of exploration on mechanism and genotype.

A study showed increased levels of TNF-α and its soluble receptor were linked to a greater decline in muscle mass and strength, but there were no differences in either IL-6 or CRP levels [61]. Besides, TNF-α was significantly increased in the frail older adults [59]. In addition, in patients with mild cognitive impairment and mild-moderate Alzheimer’s disease, TNF-α but not IL-6 contributes to an increased risk of frailty [62]. Another study from the English Longitudinal Study of Ageing with 3160 individuals over the age of 50 suggested that a multifunctional TNF gene was involved in the frailty phenotype [63]. Increased systemic TNF-α levels were associated with a higher incidence of frailty and dependency [64]. This may be because high levels of the pleiotropic TNF-α can increase muscle catabolism [65]. These findings suggest that TNF-α and its soluble receptor have the potential to be inflammatory markers of frailty. In addition, Aguirre LE et al. compared sTNFR1, IL-6, and hsCRP by multiple regression analysis and revealed that sTNFR1 was the only independent predictor of modified physical performance testing in frail obese older adults [66]. Studies explored the relationship between TNFR1 and frailty all found an association between them [30, 46, 55, 66]. Studies investigating the relationship between TNFR2 and frailty yielded contradicting results [30, 55, 61, 67].

Based on a review of available studies, IL-6, CRP, and TNF-α are consistent biomarkers of frailty [68–70]. However, it should be noted that CI is never one inflammatory mechanism acting independently, and the regulation of a single pathway has limited impact on CI status in the circulation. In preclinical models, administration of rapamycin (a suppressor of inflammation) to IL-10KO or *nfκb1*^−/−^ mice may improve their lifespan or specific physical functions, but cannot reduce IL-6 or TNF-α levels [71, 72]. Besides, various inflammatory markers are interrelated. The complexity of the interaction of inflammatory factors in vivo has been confirmed in our previous animal experiment [73]. Selective knockout of IL-6 in IL-10KO mice, on the one hand, can reverse the CI-related changes and improve the short-term functional performance of mice, while on the other hand, it also increases their mortality [73]. From current understanding of the aforementioned markers, we propose that the available candidate inflammatory markers have an uncertain or weak predictive role for frailty. There might not be just one single biological marker that reliably tracks the multitude of different contributors and phenotypes of physical frailty. A simple biologically-informed inflammatory index score (IIS) including IL-6 and TNFR1 was developed [IIS = 1/3 log(IL-6) + 2/3 log(sTNFR1)] [74]. Studies revealed that, among all 15 biomarkers measured, the IIS (HR 1.62, 95% CI 1.54 ~ 1.70, *p* < 0.05) might be the best predictor of 10-year all-cause mortality and had the best discriminatory power [74]. Furthermore, another study found that frailty had a stronger association with IIS than age [30]. IIS was also a good predictor of frailty and mortality in patients with end-stage renal disease [75] or aging HIV-infected and uninfected injection drug users [76]. The composition and calculation of IIS may still need to be optimized. The predictive effect of IIS on frailty in older adults needs to be confirmed by further studies in larger and more diverse populations to integrate it into clinical practice. Nonetheless, considering the above findings, we believe that IIS may be a potential marker of frailty. Thus, further studies in larger cohorts of subjects are needed to monitor the evolution of frailty and inflammatory biomarkers and prevent their progression to incapacity.

## Future perspectives

Based on the above facts, the research investigating inflammatory markers of frailty is still in an early stage, and evidence for the association between frailty and inflammaging comes mainly from cross-sectional studies. Diagnostic and therapeutic opportunity should be taken into consideration to maintain functional mobility and independence in aging populations. However, there are still many unresolved questions in this field. (1) Although many studies have determined that inflammatory markers can predict worse physical function, there is no consensus regarding a cut-off point. The data available are contradictory, and it is difficult to identify a unified cut-off point, because there is a lack of a unique operational definition of physical frailty. It is important to note that the reported findings in this review are largely based on the frailty phenotype criteria. (2) Physical frailty develops over years, and pathogenic processes may change throughout the course. However, most of the currently available biomarkers of physical frailty are only able to capture single aspects of the complicated conditions of frailty. Thus, in recent years, researchers have developed several different multivariate models of a panel of complementary biomarkers that were found to play a good recognitive and predictive role [27, 44, 74, 77, 78]. The advantage of the multivariate approach is that it can reflect the complex phenotypical and pathophysiological nature of frailty and allow the investigator to capture the different domains of the syndromes. However, the comprehensive calculation and complicated parameters limit its clinical use. An ideal biomarker should be valid, reproducible, reliable, specific, inexpensive and easily accessible. Bearing these considerations in mind, we propose the use of IIS, because, in addition to accurate and inexpensive, it has the advantage that it can be easily measured in serum with commercially available kits. (3) Frailty is multifactorial and has pathophysiological intersections with geriatric syndromes such as sarcopenia, and there are shared inflammatory biomarkers and pathways [79–81]. Attention should be paid to both the associations and differences between frailty and other geriatric syndromes, especially the longitudinal relationship of inflammatory markers with them. 4)To date, all the data regarding the relationship between inflammatory markers and frailty have been from observational studies. Given the midlife CI could promote later frailty [6], we believe that frailty could be reversed by primary intervention during the preclinical progression and disease initiation stages, while intervention at the irreversible damage stage might fail to reduce risk of frailty [82] (Fig. 1). There are several possible treatment methods, such as IL-6 and TNF-α inhibitors and exercise [83, 84]. Exercise can reduce age-related oxidative damage and chronic inflammation and improve mitochondrial function [84]. However, there have been few and controversial studies on the effects of clinical interventions on frailty and inflammatory factors, or the effects of anti-inflammatory treatments on frailty [81]. There is a need for rigorous and well-designed clinical trials using specific inhibitors or activators to confirm the role of inflammatory markers and further develop therapeutic targets for the management of frailty.

## Data Availability

No datasets were generated or analysed during the current study.
